# Fulacimstat Reduces Angiotensin II in Kidney Allografts in a Cross-Sectional Exploratory Study

**DOI:** 10.1016/j.ekir.2025.103745

**Published:** 2025-12-24

**Authors:** Johannes J. Kovarik, Tarik Shoumariyeh, Oliver Domenig, Marko Poglitsch, Hanna Tinel, Chantal Kopecky, Klaus G. Schmetterer, Marcus D. Säemann, Christopher C. Kaltenecker

**Affiliations:** 1 Division of Nephrology and Dialysis, Department of Medicine III, Medical University of Vienna, Vienna, Austria; 2 Attoquant Diagnostics GmbH, Vienna, Austria; 3 Department of Cardiovascular Research, Research and Development, BAYER AG, Wuppertal, Germany; 4 School of Chemistry, Australian Centre for NanoMedicine (ACN), University of New South Wales, Sydney, Australia; 5 Department of Laboratory Medicine, Medical University of Vienna, Vienna, Austria; 6 Sixth Medical Department With Nephrology and Dialysis, Clinic Ottakring, Vienna, Austria; 7 Faculty of Medicine, Sigmund-Freud University, Vienna, Austria; 8 Department of Pathology, Medical University of Vienna, Vienna, Austria

**Keywords:** ACE, angiotensin II, chymase, kidney allografts, kidney biopsy, transplantation

## Abstract

**Introduction:**

The nephroprotective effects of renin-angiotensin system (RAS) blockade after kidney transplantation (KTx) remain ambiguous. It has been shown that chymase and not angiotensin (Ang)-converting enzyme (ACE) is the most efficient Ang II–forming enzyme. Here, we investigated the efficacy of the novel and highly selective chymase inhibitor fulacimstat (BAY 1142524) on Ang II formation in human allograft biopsy tissue.

**Methods:**

In this cross-sectional, exploratory single-center study we analyzed biopsy samples of KTx recipients (*n* = 55) and healthy kidney donors (*n* = 13) with and without therapeutic RAS blockade. Using a mass spectrometry–based approach and using specific enzyme inhibitors, we performed metabolic assays to study enzyme activities of ACE and chymase and their specific contribution to intrarenal Ang II formation.

**Results:**

In contrast to healthy kidneys, a distinct shift from ACE toward chymase-dependent Ang II formation was observed in aged (> 2 years) kidney allografts. Irrespective of RAS blockade, we demonstrated high efficacy of fulacimstat (BAY 1142524) to inhibit endogenous chymase-dependent Ang II formation in biopsy tissues of human kidney allografts.

**Conclusion:**

Chymase is the key enzyme for Ang II production in aged graft vintage (> 2 years). Selective inhibition of tissue-specific chymase with fulacimstat (BAY 1142524) inhibits Ang II formation in human kidney allografts, indicating potential therapeutic effects.

## Graphical abstract

**Figure:**
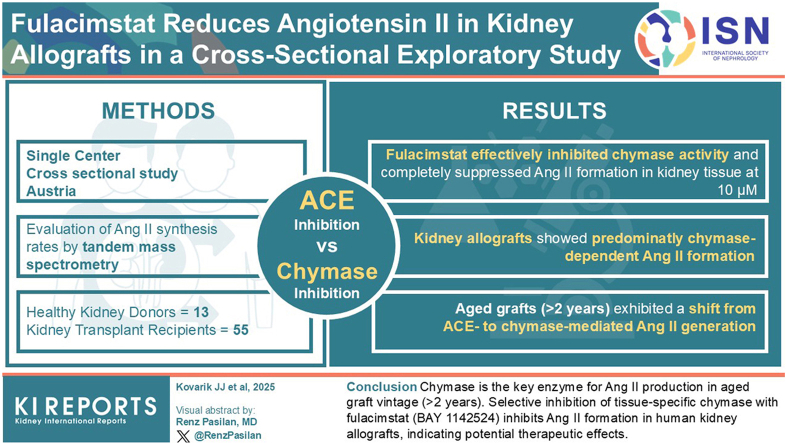

The RAS plays a central role in the regulation of blood pressure, sodium retention, and fluid homeostasis via its main effector peptides Ang II and Ang-(1–7).^1^ Pharmaceutical RAS blockade with ACE inhibitors (ACEis) and Ang receptor blockers is considered a standard therapy for patients with high blood pressure, type 2 diabetes mellitus, heart failure, and chronic kidney disease (CKD), yet is also regularly employed as antihypertensive treatment after organ transplantation. However, it has been shown that RAS blockade can only dampen but not inhibit the progression of CKD in allograft recipients.^2^ The use of RAS inhibitors after KTx remains controversial^2^ with ambiguous evidence regarding the impact on both patient and graft survival.^3^^,^^4^ Indeed, several studies have failed to demonstrate a clear beneficial effect regarding these hard clinical end points in response to RAS blockade after KTx.^5^, ^6^, ^7^, ^8^

The underlying reasons for this lack of therapeutic efficacy of RAS blockers in this patient population are still not fully elucidated. Apart from competing molecular effects stemming from immunosuppression, chronic allograft rejection, or bacterial and viral infections, the intrarenal production and regulation of both RAS effectors, Ang II and Ang-(1–7) could constitute an important factor contributing to progressive renal failure and allograft injury.^9^

One of the emerging key players of the “classical” RAS axis is the enzyme, chymase, which is a chymotrypsin-like serine protease secreted by degranulated mast cells in response to tissue injury but is also present in other cells such as cardiomyocytes.^10^^,^^11^ Chymase is the most efficient Ang II–synthesizing enzyme and can directly stimulate profibrotic signaling pathways, including transforming growth factor–β and matrix metalloproteinases,^12^ thereby affecting tissue remodeling in many renal pathologies, including CKD,^13^^,^^14^ polycystic kidney disease, diabetic kidney disease,^15^^,^^16^ and KTx rejection.^17^ Therefore, selective chymase inhibition alone or in addition to conventional RAS blockade could augment the clinical efficacy of RAS inhibition to decrease the risk of cardiovascular morbidity and mortality, particularly in conditions associated with fibrotic tissue damage such as CKD, chronic allograft damage, or after myocardial infarction.^12^^,^^18^ Our previous studies have demonstrated high degrees of ACE-independent local Ang II synthesis in CKD^14^ as well as in human allograft recipients.^19^^,^^20^ In addition, there is evidence that Ang II stimulates superoxide production,^21^ resulting in the secretion of proinflammatory cytokines such as tumor necrosis factor–α, interleukin–1β, and interleukin–6.^22^^,^^23^ Chymase is able to activate transforming growth factor–β1 independent of Ang II,^24^ which is reported to be a central factor in renal fibrosis.^25^^,^^26^ This underlines the potential of selective chymase inhibition as an attractive therapeutic strategy to interfere with ACE-independent Ang II production as well as potential Ang-independent profibrotic processes.

Fulacimstat is a potent and selective chymase inhibitor that blocks the generation of profibrotic chymase-dependent factors such as Ang II, ET-1, and transforming growth factor–β1 *in vitro* with a nanomolar half-maximal inhibitory concentration (IC_50_). The compound is highly selective and does not affect other proteases at therapeutic doses.^27^^,^^28^

The aim of this study was to determine the efficacy and functionality of the novel chymase inhibitor, fulacimstat on Ang II synthesis in human kidney allografts. We performed *ex vivo* analyses of kidney biopsy tissue employing mass spectrometry–based metabolic assays^29^ to investigate the extent of fulacimstat on Ang II generation in biopsy tissue obtained from human kidney transplant recipients and healthy kidney donors. We demonstrated the high potency of fulacimstat to inhibit endogenous chymase-dependent Ang II formation in kidney biopsy tissues. The potential relevance of our findings for future RAS targeting therapies in the transplant population is discussed.

## Methods

### Patient Recruitment

This exploratory pilot study was approved by the ethics committee of the Medical University of Vienna (EC1496/2014) and prior written informed consent was obtained from all participants. The study was conducted according to good clinical practice guidelines of the Medical University of Vienna. Kidney transplant recipients scheduled for indication biopsy due to graft dysfunction, proteinuria and/or detection of donor-specific antibodies using single antigen tests (One Lambda, Canoga Park, CA; MFI threshold: > 1000), were recruited at the outpatient clinic of the Division of Nephrology and Dialysis at the Vienna University Hospital (AKH Wien) between 2016 and 2019 (Supplementary Figure S1).

Allograft biopsies were performed under ultrasound guidance using a 16 G needle (2 cores per biopsy). Within 2 minutes, cores were evaluated and a small fragment (2 mm length) of 1 core was separated, snap-frozen in liquid nitrogen and stored at −80 °C for RAS metabolic assay. Ang II synthesis rates in biopsy tissue of KTx recipients (*n* = 55), and healthy kidney donors (*n* = 13) were measured by tandem mass spectrometry (Patient demographics in Table 1). Living kidney donors were biopsied at the time of organ explantation. Patients were grouped by allograft vintage and by use of ACEis. Blood pressure and immunosuppressive agents were recorded at the time of sample collection.

**Table 1:** Patient demographics

Biopsy group	Early KTx (*n* = 13)	Intermediate KTx (*n* = 22)	Late KTx (*n* = 20)
Age (yrs)	57 ± 9	53 ± 17	59 ± 11
Male sex (%)	64	53	59
Systolic blood pressure (mm Hg)^a^	129 [123–144] (*n* = 9)	136 [126–146] (*n* = 15)	136 ± 17 (*n* = 17)
Diastolic blood pressure (mm Hg)^a^	78 [67–83] (*n* = 9)	81 ± 13 (*n* = 15)	78 ± 10 (*n* = 17)
CKD stage (%)			
1 or 2	36	11	9
3	36	47	59
4	10	42	32
5	18	0	0
Immunosuppressive agents (%)			
Corticosteroids	91	100	77
MMF	82	79	86
Tacrolimus	91	74	14
Histopathological examination (%)			
No signs of rejection	64	74	82
Mild signs of rejection	27 (Banff BL)	11 (Banff BL)	0 (Banff BL)
Severe signs of rejection	9 (Banff 2A)	15 (Banff 2A)	13 (Banff 2A)
Insufficient material	0	0	5

CKD, chronic kidney disease; KTx, kidney transplantation; MMF, mycophenolate mofetil.

Data are shown as mean ± SD or median [inter-quartile range].

Histopathological examination was carried out according to Banff 2013 guidelines (KTx). Biopsies of healthy kidney donors showed minor acute tubular damage which is explainable by the transplantation process. Three patients showed low-grade arteriosclerosis.

a Blood pressure was not documented in all patients.

### Ang II Synthesis in Kidney Tissue

Ang II synthesis, resident ACE and chymase activity was measured in kidney tissue extracts by enzymatic addition of the natural substrate Ang I in the absence (solvent) or presence (lisinopril only / lisinopril + fulacimstat) of specific inhibitors for ACE (lisinopril) and chymase (fulacimstat), respectively. Lisinopril at 10 μM concentration assured full blockade of ACE in the assay to investigate chymase activity only. Briefly, kidney tissue was sampled by biopsy, snap frozen in liquid nitrogen, and stored at −80 °C until analysis. Tissue was dissolved in phosphate buffered saline on ice by low energy sonication (Sonics & Materials Inc, Danbury, CT). Protein content was determined using Bradford analysis and normalized to bovine serum albumin. Parallel incubation of tissue extracts (10 μg/ml) with Ang I in the absence and presence of specific inhibitors at 37 °C for 1 hour resulted in enzyme-specific Ang II formation rates presented in ([ng Ang II/μg protein]/h). Aminopeptidase inhibitors, ZPP, and MLN-4760 (all 10 μM, Sigma Aldrich) were present in all samples, assuring substrate and product stabilization. Stable isotope-labeled internal Ang standards were added for standardization and Ang II was extracted using solid phase extraction prior to liquid chromatography coupled with tandem mass spectrometry analysis. For dose response analysis of fulacimstat and chymostatin, recombinant human chymase (Sigma Aldrich) was used. Ang II was quantified using liquid chromatography coupled with tandem mass spectrometry (XEVO-TQ-S, Waters, Milford, MA) conducted by Attoquant Diagnostics GmbH, Vienna, Austria as described previously.^29^^,^^30^ Recombinant enzymes of chymase and ACE (R&D Systems, MN) were applied as quality control samples.

The enzyme-specific Ang II formation rates (*F*) were calculated as follows:

The ACE-dependent fraction of Ang II formation was calculated from the difference of synthesis rates in the absence (S_solvent_) and presence of the ACEi lisinopril (S_lisinopril_), expressed in percent of the solvent sample.

$$F_{ACE}=\frac{\left(S_{solvent}-S_{lisinopril}\right)}{S_{solvent}}$$

The residual fraction, comprising non-ACE– and non-chymase-mediated Ang II synthesis, was calculated as follows:

$$F_{residual}=\frac{\left(S_{lisinopril+fulacimstat}\right)}{S_{solvent}}$$

The chymase-dependent fraction was calculated by subtracting the above 2 fractions from 1 as follows:

$$F_{chymase}=1-\left(F_{ACE}+F_{residual}\right)$$

Due to the exploratory design of the study, only descriptive statistics were performed.

### Statistical analysis

Significant differences between Ang II synthesis rates of the different enzymes in every patient cohort were determined using 1-way analysis of variance for normally distributed variables and Kruskal–Wallis testing for non-normally distributed variables. A *P*-value < 0.05 was considered significant. GraphPad Prism version 10.2.0 (GraphPad Software, San Diego, CA) was used for statistical analysis.

## Results

### Inhibition of Ang II Synthesis With Fulacimstat and Chymostatin

**Figure 1:**
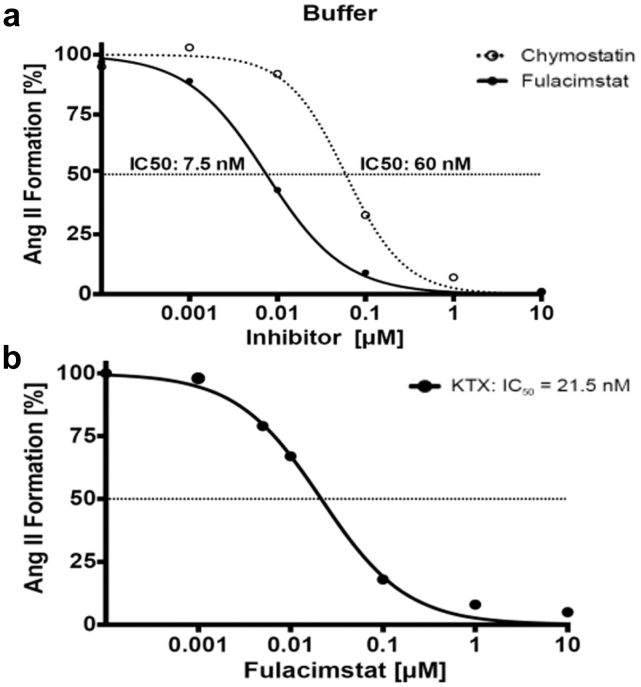
(a) Angiotensin I to angiotensin II conversion by recombinant human chymase applying the indicated chymase inhibitor concentrations in phosphate-buffered saline buffer. Reaction conditions: 1 ng/ml chymase in phosphate-buffered saline buffer, 10 μM angiotensin-converting enzyme inhibitor (lisinopril) and fulacimstat (full line) or chymostatin (dotted line). (b) Angiotensin II formation in biopsy tissue homogenates under varying inhibitor concentrations. Reaction conditions: pooled kidney tissue (*n* = 10) homogenate + 10 μM angiotensin-converting enzyme inhibitor (lisinopril) and fulacimstat. Pooled tissue was of kidney transplant recipients (with: *n* = 5; without: *n* = 5) angiotensin-converting enzyme inhibitor therapy. Ang II, angiotensin II; IC_50_, half maximal inhibitory concentration; KTx, kidney transplantation.

For dose response analysis of fulacimstat in comparison with chymostatin, we used recombinant human chymase (1 ng/ml) to process the added natural substrate Ang I in phosphate-buffered saline and measured Ang II production at varying inhibitor concentrations. Ang I to Ang II conversion by chymase in the presence of a conventional ACEi (lisinopril; 10 μM), chymostatin and fulacimstat (each ranging from 0 to 10 μM) is shown in Figure 1a. Both compounds showed comparable inhibitory capacity at the highest dose (10 μM). Fulacimstat exhibited an 8-fold lower IC_50_ (7.5 nM), indicating higher Ang II inhibitory potency than chymostatin (IC_50_, 60 nM). Thus, fulacimstat was able to block chymase activity at physiological pH and concentrations.

### Inhibition of Tissue-Resident Chymase With Fulacimstat

Next, we investigated the inhibitory potential of fulacimstat in pooled, homogenized kidney allograft biopsy samples. Ang I to Ang II conversion by endogenous tissue enzymes was determined in renal tissue in the presence of ACEi (lisinopril, 10 μM) and fulacimstat (0 to 10 μM) (Figure 1b). IC_50_ was determined to be 21.5 nM. Interestingly, we found that 10 μM fulacimstat compared with 1 μM led to a further reduction just by 3% (Table 2). At a concentration of 10 μM fulacimstat a complete inhibition of Ang II synthesis was observed.

**Table 2:** Lisinopril and fulacimstat sensitive fraction in kidney transplant recipients and healthy kidney tissue

Biopsy group (pooled samples, n=3 per group)	Lisinopril-sensitive (10 μM)	Fulacimstat-sensitive (1 μM)	Residual fraction at 1 μM fulacimstat	Residual fraction at 10 μM fulacimstat
KTx (%)	24	73	< 4	< 1
Healthy kidney donors (%)	80	14	< 5	< 2

KTx, kidney transplantation.

Pooled samples of KTx, and healthy kidney donors. Lisinopril (10 μM) and fulacimstat (1 μM) sensitive fractions were analyzed respectively.

### Chymase-Dependent Ang II Formation in Healthy Human Kidney Tissue and Allografts

**Figure 2:**
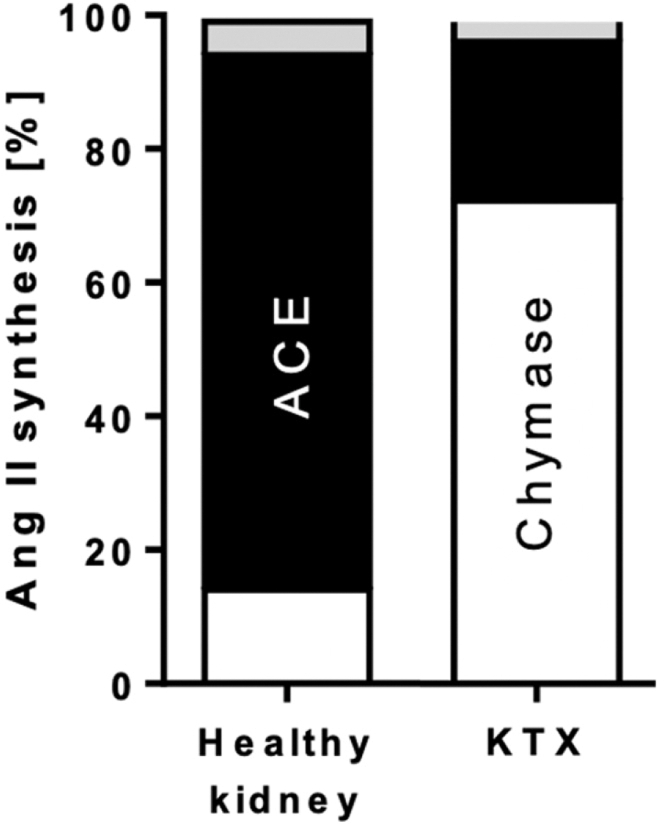
Ang II formation in pooled human tissue samples of healthy kidneys and KTx recipients. Ang II synthesis from Ang I by tissue-resident enzymes upon homogenization at pH of 7.4. Inhibitor-sensitive fractions (1 μM fulacimstat and 10 μM lisinopril) of chymase (in white) and ACE (in black) toward Ang II synthesis (inhibitor-insensitive part in grey; residual fraction) in pooled samples of healthy kidneys (*n* = 3) and kidney transplant recipients (*n* = 3). ACE, angiotensin converting enzyme; Ang, angiotensin; KTx, kidney transplantation.

We observed complete inhibition of Ang II formation in the investigated kidney tissue homogenates with a concentration of 1 μM fulacimstat (Figure 1b). Further analysis of tissue-specific ACE and chymase contribution to Ang II formation in healthy kidneys and kidney allografts was then performed. Samples were treated with ACEi (lisinopril, 10 μM) in addition to 1 μM fulacimstat (Figure 2). Kidney transplant recipients showed high chymase dependence (> 70%) in local Ang II synthesis. In contrast, ACE was found to be the dominant enzyme for Ang II formation in healthy kidneys.

### Enzymatic Processing of Ang I to Ang II in Kidney Transplant Recipients With Different Graft Vintage

**Figure 3:**
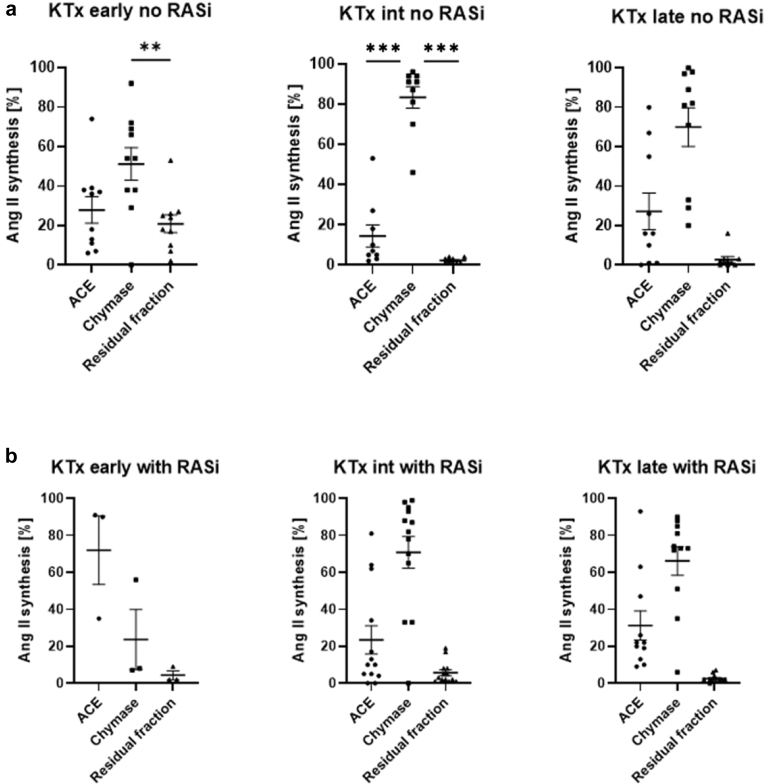
Ang II synthesis in kidney transplant recipients. (a) Relative contribution (%) of ACE, chymase, and residual fraction toward Ang II synthesis in patients without RAS blockade (no RASi). Scatter plots grouped according to KTx vintage (KTx early: < 2 years [*n* = 10], KTx intermediate: 2–12 years (*n* = 9), KTx late: > 12 years (*n* = 9). (b) Relative contribution (%) of ACE, chymase, and residual fraction toward Ang II synthesis in patients with RAS blockade (with RASi). Scatter plots grouped according to allograft vintage (KTx early: < 2 years [*n* = 3], KTx intermediate: 2–12 years (*n* = 13), KTx late: > 12 years (*n* = 11). ∗*P* < 0.05; ∗∗*P* < 0.001, ∗∗∗*P* < 0.0001. ACE, angiotensin converting enzyme; Ang II, angiotensin II; Int, intermediate; KTx, kidney transplantation; RASi, renin angiotensin system inhibitor.

Next, the relative contribution of inhibitor-sensitive fractions of chymase and ACE toward Ang II synthesis was analyzed. Biopsy samples were grouped according to graft vintage (< 2 years, 2–12 years, > 12 years) and according to the patients’ existing ACEi therapy (Figure 3a and b, Table 3). Ang II synthesis in kidney transplant recipients within the first 2 years after KTx showed lowest fulacimstat sensitivity (8%–54%) compared with aged allografts (> 2 years). Kidney transplant recipients early after KTx (< 2 years) receiving ACEi therapy showed high local ACE activity compared with those without RAS blockade, and to kidney transplant recipients with a higher graft vintage (> 2 years) with or without ACEi therapy. In addition, Ang II formation by chymase is significantly higher in aged graft vintage (> 2 years) in patients which did not receive RAS blockade (Figure 3a). In patients receiving RAS blockade, there was a significantly higher Ang II formation by chymase only in allografts with a vintage of 2 to 12 years (Figure 3b).

**Table 3:** Ang II synthesis rates (%) of analyzed patient cohorts

	Sample size	KTx vintage	Ang II synthesis rates [%]			*P*-value
			Lisinopril-sensitive (ACE)	Fulacimstat-sensitive (chymase)	Residual fraction (RF)	
KTx recipients without RASi						
	10	< 2 yrs	27.9 ± 21.1	51.2 ± 26	20.9 ± 14.2	ACE vs. chymase: 0.05 ACE vs. RF: 0.74 Chymase vs. RF: 0.01
	9	2–12 yrs	14.4 ± 16.6	83.3 ± 16.2	2.2 ± 1.3	ACE vs. chymase: < 0.0001 ACE vs. RF: 0.15 chymase vs. RF: < 0.0001
	9	> 12 yrs	16 [1–58]	81.5 [32–97.3]	1.5 [0–3]	0.0001
KTx recipients with RASi						
	3	< 2 yrs	90 [35–91]	8 [7–56]	2 [2–9]	0.08
	13	2–12 yrs	10 [4.5–48]	82 [49–94]	3 [1.5–8]	0.0001
	11	> 12 yrs	22 [13–47]	73 [51–85]	2 [1–4]	< 0.0001
Healthy kidney donors without RASi						
	7		90 [69–91]	9 [8–13]	2 [1–15]	< 0.0001
Healthy kidney donors with RASi						
	6		89 [66–91]	8 [6–33]	3 [2–3]	< 0.0001

ACE, angiotensin converting enzyme; Ang II, angiotensin II; KTx, kidney transplantation; RASi, renin-angiotensin system inhibitors; RF, residual fraction.

Ang II synthesis rates reported as mean +/- SD in normally distributed cohorts and as median [interquartile range] in non-normally distributed study cohorts. *P*-values are reported in every enzyme cohort in normally distributed study cohorts (analysis of variance) and in non-normally distributed study cohorts an overall *P*-value has been reported (Kruskal-Wallis-Test).

### Immunohistochemical and Clinical Correlation

To evaluate whether chymase activity is associated with mast cell infiltration, we performed a correlation analysis, including 13 data points. This analysis revealed no significant correlation (Supplementary Figure S2).

We further examined the potential relationship between fulacimstat-sensitive Ang II synthesis and clinical parameters, namely estimated glomerular filtration rate and urinary protein-to-creatinine ratio. Correlation analyses showed no evidence of an association in either case (Supplementary Figure S3 and S4).

## Discussion

Pharmacological RAS blockade constitutes the cornerstone of antihypertensive therapy in patients with CKD.^31^ However, emerging evidence questions the beneficial effects of RAS inhibition in kidney transplant recipients, indicating a limited efficacy of these drugs on graft and patient survival in this population.^2^^,^^6^ Recently, we and others showed that high local Ang II synthesis is mediated by chymase in CKD^2^^,^^4^ and transplanted heart and kidney tissue.^14^^,^^19^^,^^20^^,^^32^ However, chymase is not targeted by conventional RAS blockade. Our study was aimed at elucidating the effects of fulacimstat on Ang II formation in biopsies of human kidney allografts. We grouped the biopsies into 3 groups according to their allograft vintage. By performing highly sensitive mass spectrometry-based metabolic assays,^19^^,^^30^ we could demonstrate within this study the effective *ex vivo* inhibition of tissue Ang II synthesis by the novel potent and specific chymase inhibitor fulacimstat in human kidney transplant recipients.

Our findings of high chymase activity in kidney allografts are in line with a report from Wasse *et al.*^33^ showing an association of chymase bearing mast cell density with tubulointerstitial fibrosis severity in diverse renal pathologies, including allograft rejection.

Furthermore, it has previously been shown that high formation of local Ang II may cause renal injury via its ability to induce vasoconstriction and ischemia.^8^ Recent evidence points toward a high ACE-independent enzyme contribution to local Ang II formation in diseased hearts and kidneys^19^^,^^20^ which is associated with chronic inflammation, diabetes, as well as chronic kidney allograft rejection and overall allograft damage.^14^^,^^17^^,^^34^^,^^35^

Therefore, the concept of therapeutic chymase inhibition might provide a feasible complementary approach to conventional RAS blockade to control the detrimental actions of Ang II in acute and chronic diseases.^12^ However, although chymase inhibition was safe and well-tolerated, it neither reduced albuminuria in patients with diabetic kidney disease nor influenced cardiac remodeling in patients with left-ventricular dysfunction after first myocardial infarction.^32^^,^^36^

Despite the lack of positive effects of chymase inhibition in these studies, it does not preclude beneficial clinical effects in general. However, future prospective randomized long-term clinical trials will have to elucidate the full potential of this therapeutic approach.

Because no data of chymase inhibitors in the transplant population are published, we studied the effects of the novel chymase inhibitor fulacimstat on Ang II formation in biopsy tissue of human allografts compared to healthy kidney tissue.

We observed a distinctly lower IC_50_ of fulacimstat than of chymostatin, indicating a higher potency of this novel compound *in vitro.* Chymostatin is a protease inhibitor which inhibits chymase and other enzymes such as chymotrypsin, cathepsin G, and papain, as well as cysteine proteases.^37^ Thus, for the experiments with tissue homogenates, we preferred to use the selective chymase inhibitor, fulacimstat instead of chymostatin. Fulacimstat selectively inhibits chymase and is not influencing the activity of other proteases except for cathepsin G which is inhibited with 35-fold lower potency compared with chymase.^38^ Interestingly, we found no significant differences in enzyme contribution to Ang II formation between patients with or without conventional RAS blockade in any of the analyzed tissues in this study. An exception was biopsy tissue of kidney transplant recipients with ACEi therapy, early after KTx (< 2 years), in which we observed a high ACE activity. This leads to the assumption that our observation of high ACE activity in healthy kidneys could, at least partly, still be present in kidney allografts in the early period after KTx. Although ACE is the predominant Ang II–forming enzyme in healthy kidneys, chymase is directing Ang II generation, particularly in aged allografts (> 2 years) (Figure 3a). However, because the patients were not randomized for treatment, and sampling time is dependent on kidney function, the data has to be viewed as exploratory. Therefore, we cannot make statements about the dynamics of the Ang II production throughout the posttransplant time. Owing to the exploratory design of the study, detailed analysis of Ang II synthesis of patients with Ang receptor blocker therapy compared with ACEi therapy was not performed within this study and should be analyzed in future studies. In addition, the influence of factors such as kidney donor age, human leukocyte antigen matching, preformed and course of donor-specific antibodies on Ang II formation should be analyzed in detail in the future (Supplementary Discussion).

Nevertheless, these and previous results of our research group suggest that the shift from ACE to chymase takes place in the first 2 years after KTx.^20^ This shift in kidney RAS activity was reflected by the high count of chymase-containing mast cells in long-term kidney transplants.^20^ However, we did not observe any conclusive correlation between biopsy findings and outcomes of our RAS analysis. Because the majority of our analyzed transplant patients had no allograft rejection, we cannot draw any conclusions to which extent transplant rejection affects chymase (e.g., via mast cell infiltration).

Nevertheless, several studies have exemplified the potential of mast cells and chymase in determining the cardiorenal risk profile in heart disease by RAS-associated and RAS-independent action.^39^, ^40^, ^41^ Accumulating evidence suggests a fundamental role of mast cell clustering and subsequent chymase release in inflammatory pathologies and tissue remodeling.^12^ The novel view of chymase as a key RAS enzyme for kidney remodeling, significantly contributing to tissue Ang II formation urgently requires further research. Detailed elucidation of the pivotal role of this enzyme in human allografts with special regard to progression of tissue fibrosis is urgently required. Therefore, it might be of high interest to evaluate the role of chymase inhibitors in the transplant population.

The present study has some limitations. First, enzymes such as chymase are liberated from their cellular compartments by tissue homogenization, thus potentially overestimating their influence on Ang II formation in our samples. Second, we only analyzed the RAS effector Ang II and its generating enzyme pathways, and we have not investigated the effects of fulacimstat on further downstream Ang pathways, including the nephroprotective Ang-(1–7). Moreover, we cannot rule out that some other enzymes such as cathepsin G might contribute to the Ang II generation. At high concentrations, cathepsin G activity might be slightly reduced by fulacimstat. Third, this study has used fulacimstat exclusively for *in vitro* assays and caution is warranted to fully translate these findings directly to *in vivo* models or therapeutic use; for example, because of pharmacokinetic or pharmacodynamic properties of the compound. Furthermore, we are aware of the fact that immunosuppressive agents, such as calcineurin inhibitors, may affect the renin content of renal cells, yet we did not perform a randomized controlled trial to investigate this specifically. In addition, an important limitation of this study is the relatively small sample size, which reduces statistical power. Although the observed shift from ACE- to chymase-dominant Ang II formation in aged kidney allografts remained consistent across multiple assays, the limited number of samples restricted our ability to explore potential confounders in greater depth. The performance of multivariate analyses that could account for factors such as donor comorbidities, ischemia-reperfusion parameters, immunosuppressive regimens, and chronic allograft injury scores was not possible because of the small sample size. Because of the small sample size, descriptive statistics have been used, which could influence the robustness of the findings given that descriptive statistics cannot establish causality and are sensitive to outliers. Therefore, the results should be interpreted as exploratory rather than definitive. Future studies with larger, prospectively collected cohorts will be essential to validate these findings and determine the relative contribution of chymase activity within the broader context of kidney allograft aging and remodeling.

The strengths of this study are the analyses of the following: (i) human kidney biopsy samples and (ii) using Ang I as a natural substrate to profile Ang II formation by tandem-mass spectrometry.^29^^,^^30^ The applied Ang synthesis assays represent an innovative tool to advance our current understanding of the molecular effects of standard RAS blockers as well as novel RAS-interfering drugs such as chymase inhibitors, combined Ang I receptor blockers, and neprilysin inhibitors.^42^ This substantially advances our understanding of RAS regulation and helps to optimize personalized RAS blockade in kidney transplant recipients. Previously, endogenous Angs were quantified in rodent kidneys using mass spectrometry^29^^,^^43^; however, this approach cannot be replicated because of the limited sample amount obtainable from kidney biopsies. We have overcome this issue by analyzing tissue enzyme activities to characterize kidney RAS metabolism.^19^^,^^34^

The observed high proportion of chymase-dependent Ang II production in kidney allografts could indicate that chymase might be a promising therapeutic target to dampen the progression of allograft fibrosis and its deleterious consequences on allograft function and survival. Nevertheless, further studies are needed to evaluate clinical implications of chymase inhibition in kidney transplant recipients.

In conclusion, we demonstrated that fulacimstat potently inhibits endogenous chymase-dependent Ang II formation in kidney biopsy tissues. These *ex vivo* findings provide preliminary support for further investigation of chymase inhibition as a potential therapeutic approach in KTx. However, the translational relevance of these observations remains to be established. Additional experimental studies, as well as well-designed clinical investigations of fulacimstat are required to determine its *in vivo* efficacy and potential role in improving graft and patient outcomes.

## Disclosure

HT is currently employed at BAYER AG. All the other authors declared no competing interests.

## Supplementary Material

Supplementary File (PDF) **Discussion Regarding the Interpretation of the Supplementary Figures and Tables. Figure S1.** Flowchart showing the distribution of the different study populations. **Figure S2.** Histological slides (1–4 slides, median 2) were stained with chymase or c-Kit double stain and double-positive cells were counted. Chymase activity shown as ng angiotensin II/μg protein/h. Mast cell count (median of 1-4 slides) shown normalized to tissue area. **Figure S3.** Kidney function on day of biopsy versus chymase-dependent angiotensin II synthesis. **Figure S4.** Urinary protein creatinine ratio [g/g] on day of biopsy versus chymase-dependent angiotensin II synthesis. **Table S1.** Distribution of donor-specific antibody presence in all patient subgroups. **Table S2.** Human leukocyte antigen mismatch sum (HLA-A, HLA-B, HLA-DR) in all patient subgroups: range 0–6. **Table S3.** Count of deceased and living donors among all patient subgroups.

## Footnotes

Supplementary File (PDF)

**Discussion Regarding the Interpretation of the Supplementary Figures and Tables.**
**Figure S1.** Flowchart showing the distribution of the different study populations. **Figure S2.** Histological slides (1–4 slides, median 2) were stained with chymase or c-Kit double stain and double-positive cells were counted. Chymase activity shown as ng angiotensin II/μg protein/h. Mast cell count (median of 1-4 slides) shown normalized to tissue area. **Figure S3.** Kidney function on day of biopsy versus chymase-dependent angiotensin II synthesis. **Figure S4.** Urinary protein creatinine ratio [g/g] on day of biopsy versus chymase-dependent angiotensin II synthesis. **Table S1.** Distribution of donor-specific antibody presence in all patient subgroups. **Table S2.** Human leukocyte antigen mismatch sum (HLA-A, HLA-B, HLA-DR) in all patient subgroups: range 0–6. Data shown as median [inter-quartile range] **Table S3.** Count of deceased and living donors among all patient subgroups.
