# Supplementary material for: Fulacimstat Reduces Angiotensin II in Kidney Allografts in a Cross-Sectional Exploratory Study
Source: Kidney Int Rep. 2025 Dec 24;11(3):103745. doi: 10.1016/j.ekir.2025.103745 (PMC12857369; doi:10.1016/j.ekir.2025.103745)
Supplement: Supplementary File (PDF) — Discussion Regarding the Interpretation of the Supplementary Figures and Tables. Figure S1. Flowchart showing the distribution of the different study populations. Figure S2. Histological slides (1–4 slides, median 2) were stained with chymase or c-Kit double stain and double-positive cells were counted. Chymase activity shown as ng angiotensin II/μg protein/h. Mast cell count (median of 1-4 slides) shown normalized to tissue area. Figure S3. Kidney function on day of biopsy versus chymase-dependent angiotensin II synthesis. Figure S4. Urinary protein creatinine ratio [g/g] on day of biopsy versus chymase-dependent angiotensin II synthesis. Table S1. Distribution of donor-specific antibody presence in all patient subgroups. Table S2. Human leukocyte antigen mismatch sum (HLA-A, HLA-B, HLA-DR) in all patient subgroups: range 0–6. Table S3. Count of deceased and living donors among all patient subgroups. [file mmc1.pdf]

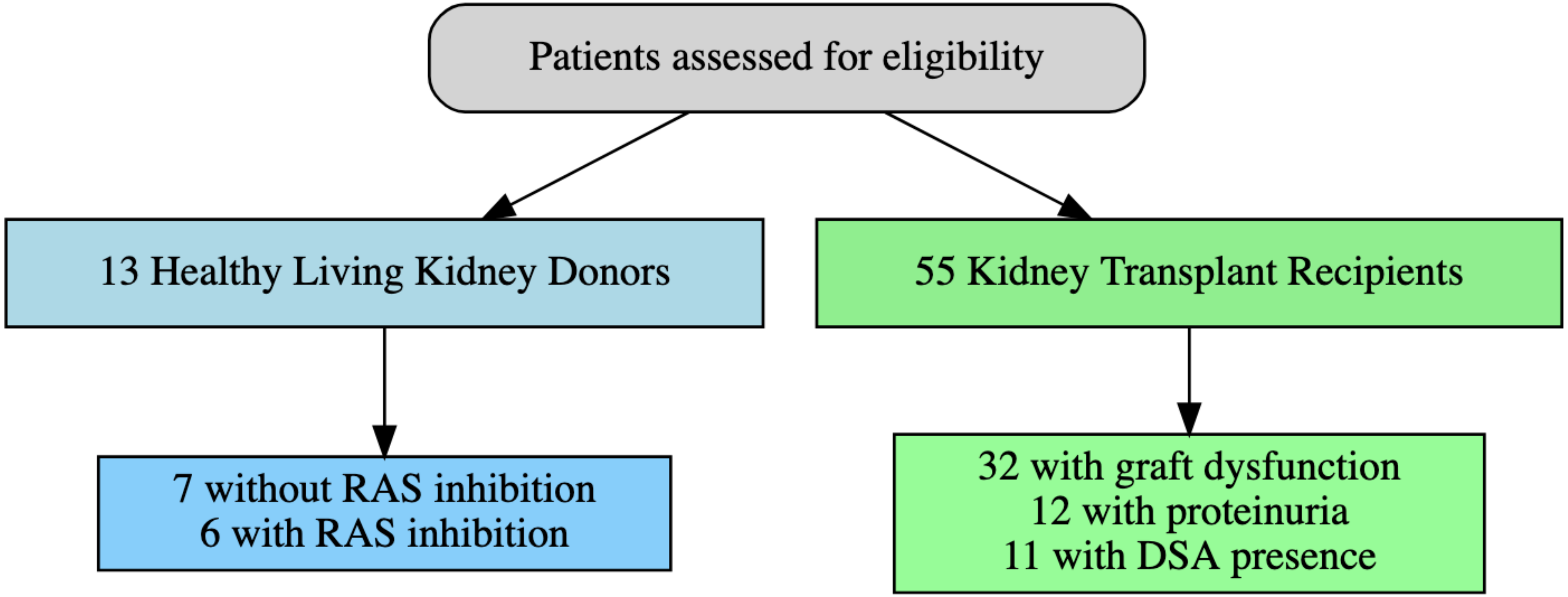

Supplementary Fig. S1. Flowchart showing the distribution of the different study populations.

DSA, donor-specific antibody; RAS, renin angiotensin system

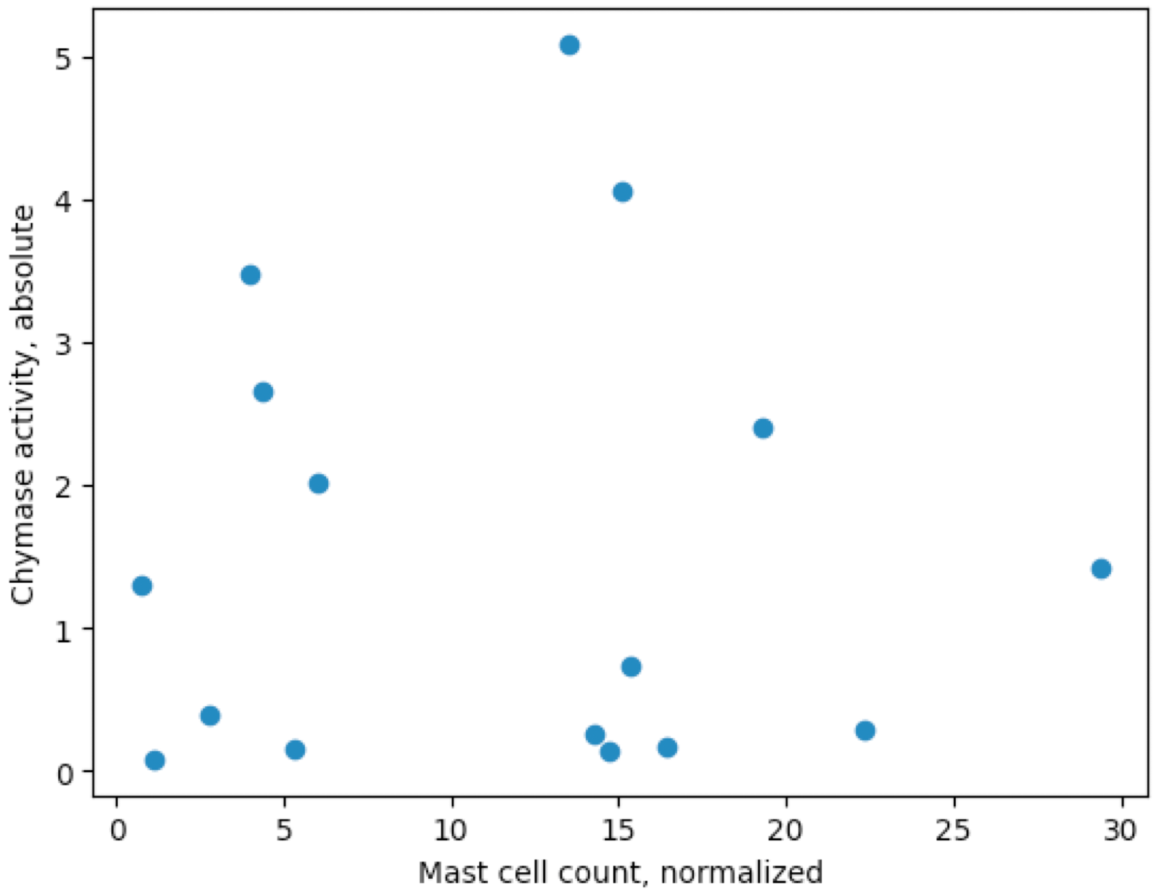

Supplementary Fig. S2. Histological slides (1-4 slides, median 2) were stained with chymase/c-Kit double stain and double-positive cells were counted. Chymase activity shown as ng Ang II/µg protein/h. Mast cell count (median of 1-4 slides) shown normalized to tissue area.

Ang II, angiotensin II

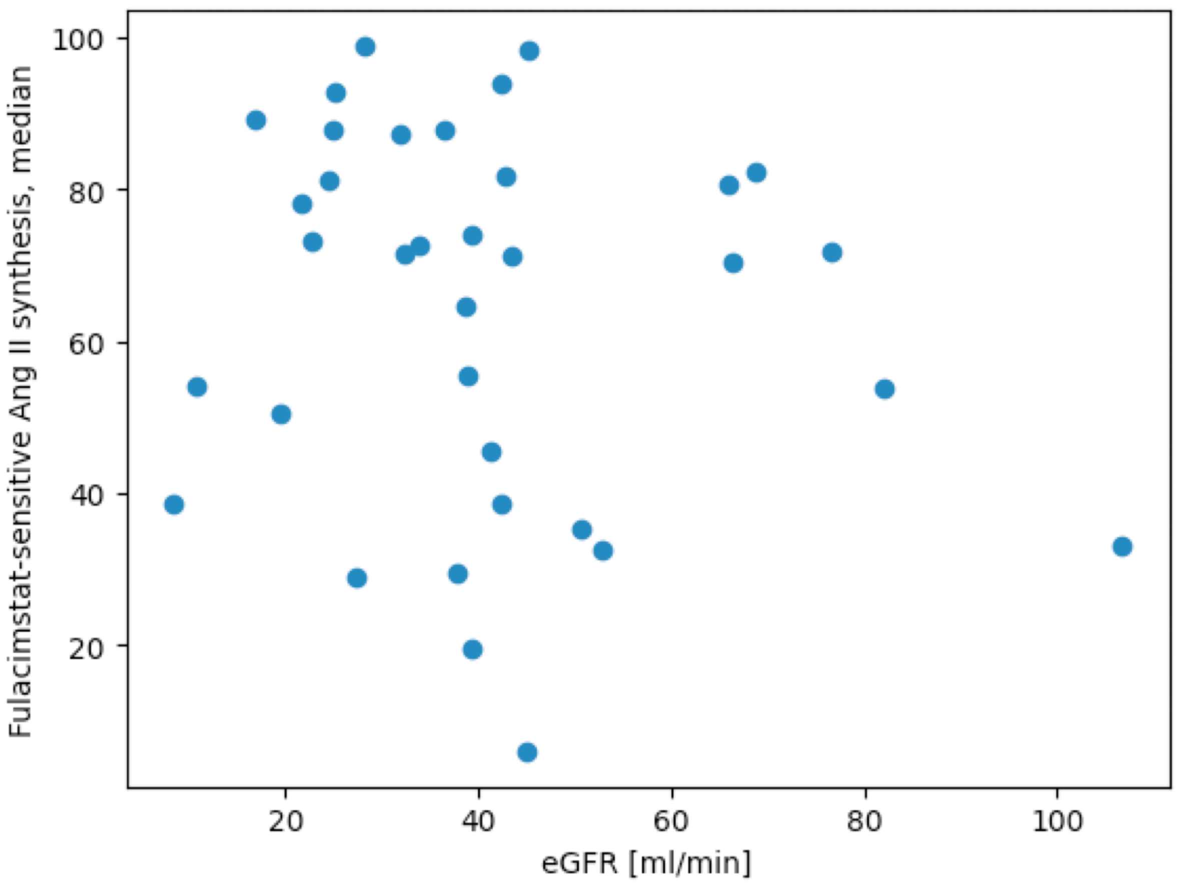

Supplementary Fig. S3. Kidney function at day of biopsy vs chymase-dependent Ang II synthesis.

Ang II, angiotensin II

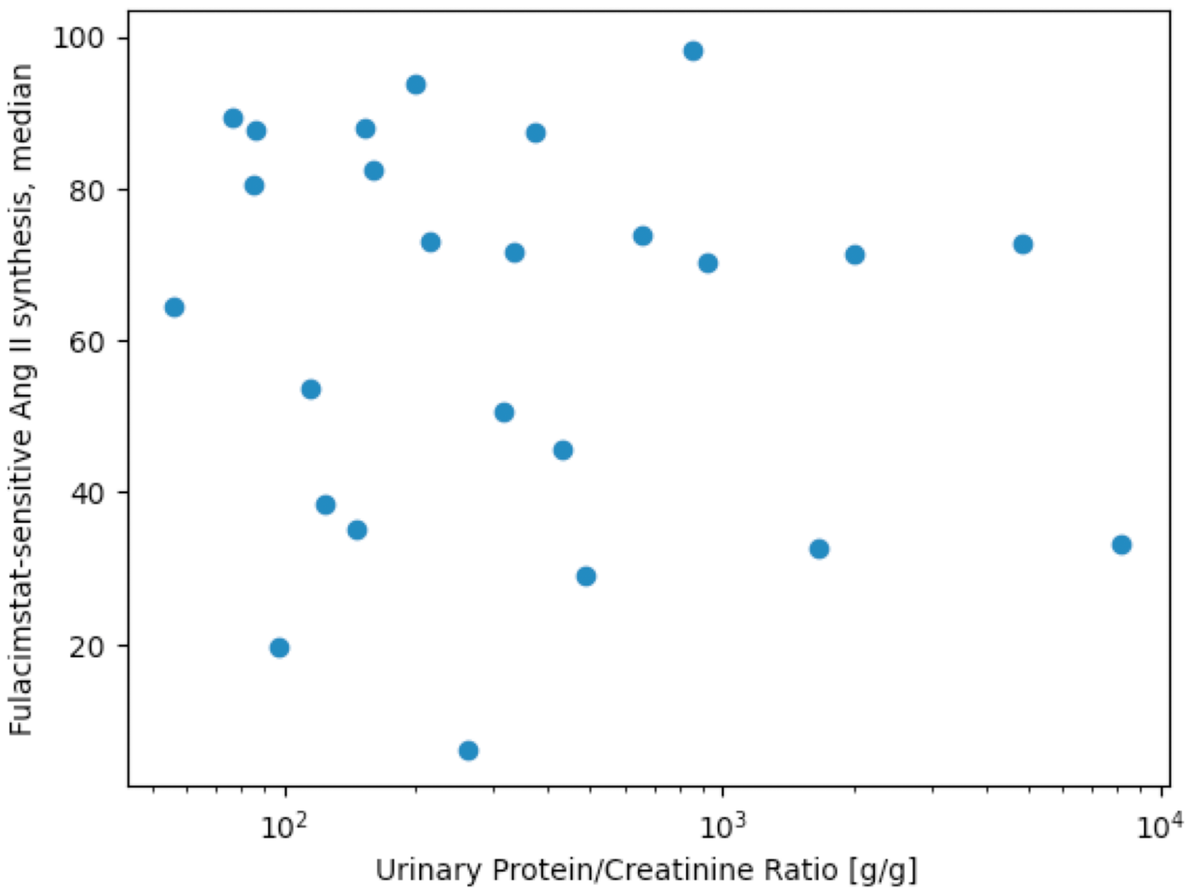

Supplementary Fig. S4. Urinary Protein Creatinine Ratio [g/g] at day of biopsy vs chymase-dependent Ang II synthesis.

Ang II, angiotensin II

	no RASi, early	no RASi, intermediate	no RASi, late	ACEi, early	ACEi, intermediate	ACEi, late
Negative	3 (38%)	1 (11%)		1 (33%)	4 (44%)	3 (43%)
Positive	3 (38%)	7 (78%)	6 (86%)	2 (66%)	3 (33%)	3 (43%)
No data	2 (25%)	1 (11%)	1 (14%)		2 (22%)	1 (14%)

Supplementary Table S1. Distribution of DSA presence in all patient subgroups. DSA, donor-specific antibody

	no RASi, early	no RASi, intermediate	no RASi, late	ACEi, early	ACEi, intermediate	ACEi, late
sum	3.5 [3–4]	3 [2–5]	3 [2.5–3]	3 [3–3.5]	3 [2–3]	3 [2–3.5]

Supplementary Table S2. HLA mismatch sum (HLA-A, HLA-B, HLA-DR) in all patient subgroups: range 0-6. Data shown as median [inter-quartile range]; HLA, human leukocyte antigen

	no RASi, early	no RASi, intermediate	no RASi, late	ACEi, early	ACEi, intermediate	ACEi, late
**No data**	5 (62%)			2 (66%)	2 (22%)	
**DD**	3 (38%)	5 (56%)	6 (86%)	1 (33%)	7 (77%)	6 (86%)
**LD**		4 (44%)	1 (14%)			1 (14%)

Supplementary Table S3. Count of deceased and living donors among all patient subgroups.

DD, deceased donor; LD, living donor

Discussion regarding the interpretation of the supplementary figures and tables.

We retrieved available data, but some data for donor type (n=9) and donor-specific antibody (DSA) presence (n=7) were missing. These data revealed DSA presence in 38-78% in respective groups, but without clear difference between the groups (i.e. the transplant vintage). Human leukocyte antigen (HLA) mismatch count (sum of HLA-A, -B, and -DR) was evenly distributed in all groups (count: 3 [2-4]). Deceased donor type was most common in all groups, whereas living donor type was observed in 6 patients only.

Owing to the small sample size, we opted for descriptive measures without statistical testing.

Nevertheless, the exposure time to these risk factors (i.e. positive DSA, high HLA mismatch, DD donor type) would be inherently different in the patient groups (since they are defined by post-transplantation time), and at the same time a potential bias (since patients with high risk factors are less likely to retain organ function) making it a vague proxy for tissue injury.

High mast cell counts were observed in patients with histological features of inflammation and antibody-mediated rejection as well as high chronic lesion scores (Banff ci >1, ct>1).
